# Isolation of a novel *Bacillus subtilis* HF1 strain that is rich in lipopeptide homologs and has strong effects on the resistance of plant fungi and growth improvement of broilers

**DOI:** 10.3389/fmicb.2024.1433598

**Published:** 2024-10-01

**Authors:** Qianru Li, Ying Wang, Chao Chen, Mingbai Zeng, Qingyun Jia, Jinhao Ding, Chenjian Zhang, Shanhai Jiao, Xupeng Guo, Jihua Wu, Chengming Fan, Yuhong Chen, Zanmin Hu

**Affiliations:** ^1^Key Laboratory of Seed Innovation, Institute of Genetics and Developmental Biology, Innovation Academy for Seed Design, Chinese Academy of Sciences, Beijing, China; ^2^College of Advanced Agricultural Sciences, University of Chinese Academy of Sciences, Beijing, China; ^3^AUSCA Oils and Grains Industries Co., Ltd., Fangchenggang, China; ^4^The 306th Hospital of PLA, Beijing, China

**Keywords:** *Bacillus subtilis*, antifungal activity, secondary metabolites, lipopeptides, feed additives

## Abstract

*Bacillus subtilis* is an important probiotic microorganism that secretes a variety of antimicrobial compounds, including lipopeptides, which are a class of small molecule peptides with important application value in the fields of feed additives, food, biopesticides, biofertilizers, medicine and the biological control of plant diseases. In this study, we isolated a novel *B. subtilis* HF1 strain that is rich in lipopeptide components and homologs, has a strong antagonistic effect on a variety of plant fungi, and is highly efficient in promoting the growth of broilers. The live *B. subtilis* HF1 and its fermentation broth without cells showed significant inhibitory effects on 20 species of plant fungi. The crude extracts of lipopeptides in the fermentation supernatant of *B. subtilis* HF1 were obtained by combining acid precipitation and methanol extraction, and the lipopeptide compositions were analyzed by ultrahigh-performance liquid chromatography with quadrupole time-of-flight mass spectrometry (UPLC-Q-TOF-MS). The results showed that HF1 could produce 11 homologs of surfactin and 13 homologs of fengycin. Among the fengycin homologs, C_13_-C_19_ fengycin A and C_15_-C_17_ fengycin B were identified; among the surfactin homologs, C_11_-C_17_ surfactin A and C_13_-C_16_ surfactin B were characterized. C_13_ fengycin A, C_11_ surfactin A and C_17_ surfactin A were reported for the first time, and their functions are worthy of further study. In addition, we found that HF1 fermentation broth with and without live cells could be used as a feed additive to promote the growth of broilers by significantly increasing body weight up to 15.84%. HF1 could be a prospective strain for developing a biocontrol agent for plant fungal diseases and an efficient feed additive for green agriculture.

## 1 Introduction

*Bacillus* spp. are unique in their formation of endospores and their ability to produce large amounts of antimicrobial compounds that facilitate their coexistence in soil, aquatic environments, and gut microbiota (Nicholson, 2002; Caulier et al., 2019), and the four original species of this microbiota (*B. subtilis*, *Bacillus licheniformis*, *Bacillus pumilus*, and *Bacillus amyloliquefaciens*) were discovered more than 40 years ago (Zeigler and Perkins, 2008). *B. subtilis* is a non-pathogenic microorganism that is nontoxic and harmless to humans and animals, environmentally friendly, and antagonistic to plant and animal pathogens (Du et al., 2019); it can be used for plant disease control and as feed additive, and resistance in target pathogens is not easily induced after its long-term use. In the biotechnology industry, *B. subtilis* is widely used as a chassis for the biosynthesis of natural products, from enzymes to bioactive compounds (Hara et al., 2014; Drejer et al., 2018). In recent years, *B. subtilis* has also gained increasing attention as a biological control agent and feed additive because it can antagonize plant pathogens, promote plant growth, and improve animal health (Mohamed et al., 2022; Qiu et al., 2021; Iqbal et al., 2021).

Fungal pathogens are important pathogens affecting crop production and food safety, and the control of plant fungal diseases is carried out mainly through chemical fungicides (Yoon et al., 2013; McLaughlin et al., 2023). However, the overuse and inappropriate use of fungicides has caused environmental disruption and health problems and increased fungal pathogen resistance to fungicides (Maltby et al., 2009; Alavanja et al., 2014). As an alternative to traditional fungal control methods, the use of biocontrol fungicides is considered a safe and environmentally friendly strategy for reducing the use of chemical fungicides. The most attractive option as a biological control agent is *B. subtilis*, whose antifungal potential has been extensively studied (Wu et al., 2021; Ling et al., 2022; Silveira et al., 2022; Zhang et al., 2022).

The mechanism by which *B. subtilis* antagonizes fungal pathogens is significantly diverse due to its different metabolites. Studies have shown that approximately 4 to 5% of the genome of wild-type *B. subtilis* is dedicated to the synthesis of bioactive metabolites (Stein, 2005). The bioactive metabolites produced by *Bacillus* spp. can be classified into five categories: nonribosomal peptides (NRPs), polyketides (PKs), ribosomal peptides (RPs), and hybrid and volatile metabolites. Lipopeptides are low-molecular-weight, amphiphilic biosurfactants that are catalyzed by nonribosomal peptide synthetase (NRPS) or polyketide synthetase (PKS) (Chen et al., 2009; Yin et al., 2024). The complex synthesis mechanisms result in lipopeptides that vary greatly in the type and sequence of amino acid residues, the nature of peptide cyclization, and the nature, length, and branching of fatty acid chains (Ben Abdallah et al., 2015), which can be divided into two categories, cyclic lipopeptides and linear lipopeptides, based on the different amino acid structures (Ortíz-López et al., 2015). According to the structural characteristics of cyclic peptides, *Bacillus* lipopeptides can be divided into three main families: fengycins (decapeptides with a lactone ring in the peptidic moiety and a *β*-hydroxyl fatty acid chain), iturins (a lactam containing a C_14_-C_17_ β-amino fatty acid attached to a heptapeptide), and surfactins (containing a cyclic lactone ring with a C_12_-C_16_ β-hydroxy fatty acid and a heptapeptide) (Yang et al., 2015; Qiao et al., 2024), and these molecules have been proven to exhibit various biological activities.

Within the surfactin lipopeptide family, there is a wide spectrum of different homologs and isomers; to date, more than 30 variants have been described. Surfactin has many biological activities, such as inhibiting plant pathogens, resisting bacteria, fungi, viruses, tumors, and mycoplasma, and anti-adhesion activity (Al-Wahaibi et al., 2014; Deravel et al., 2014; Duarte et al., 2014; Liu et al., 2010). Generally, the bactericidal activity of lipopeptides increases with increasing fatty acid chain length (usually C_10_-C_12_), and lipopeptides with a higher number of carbon atoms (14 or 16) have stronger antibacterial or antifungal activity (Baindara et al., 2013; Santos et al., 2023). The number of carbon atoms in the fatty acid chain of surfactin affects its viral inactivation ability, and surfactin shows stronger antiviral activity with the increase in the number of carbon atoms in the fatty acid chain (Kracht et al., 1999). Due to the application potential of surfactins, much work has been carried out to increase their production (Wang et al., 2020; Hu et al., 2019; Sun et al., 2009; Willenbacher et al., 2016).

Fengycin has broad-spectrum antifungal activity and is particularly effective against filamentous fungi; it has great application potential for agricultural biological control, clinical medical treatment, environmental treatment and other fields (Koumoutsi et al., 2004; Vanittanakom et al., 1986; Vargas-Straube et al., 2016). The production of fengycin can be increased by replacing the promoter, upregulating the expression of related genes involved in the fatty acid pathway, and adding exogenous amino acids (Gao et al., 2022; Tan et al., 2022).

Probiotics have attracted major interest in the livestock industry; they are defined as “nonpathogenic live microbial feed supplements,” and the addition of probiotics to diets can improve animal health and performance by promoting gut health and nutrient utilization (Grant A. Q. et al., 2018). A recent study in Nature by Piewngam et al. demonstrated that the *Bacillus* lipopeptide fengycin restricts intestinal *Staphylococcus aureus* colonization by inhibiting quorum sensing (Chung and Raffatellu, 2019). *B. subtilis* has been widely used as a feed supplement (Alagawany et al., 2018; Alagawany et al., 2019; Bahaddad et al., 2023). However, because the types and contents of metabolites produced by different *B. subtilis* strains are different, and their effects on livestock are also significantly different. Therefore, the isolation and construction of new strains that are particularly effective at controlling plant diseases or promoting animal health is a long-term goal of scientists.

In this study, we isolated an excellent strain from the rhizosphere of cucumber, HF1, which has strong broad-spectrum antifungal activity and can significantly promote the growth of broilers. We assembled the full-length genomic DNA of the strain, predicted its secondary metabolites, and identified new homologs of surfactins and fengycins in its fermentation broth. This study provides a new option for the biological control of plant fungal disease and an efficient feed additive for healthy broiler production by using strain HF1.

## 2 Materials and methods

### 2.1 Isolation and characterization of the HF1 strain

HF1 was isolated from the rhizosphere of cucumber on a farm in Beijing using the spread plate method (Sanders, 2012) and screened by its ability to resist the fungal pathogen *Alternaria alternata*, which can cause brown spots in tobacco, and grows rapidly on potato dextrose agar (PDA) medium.

### 2.2 The characterization of the physiological and biochemical properties of the HF1 strain

The physiological and biochemical characteristics of HF1 were analyzed by a variety of tests using the methods described previously (Ma et al., 2024), including anaerobic growth, sugar alcohol fermentation, starch hydrolysis, methyl red, catalase, oxidase, gelatine liquefaction, Voges-Proskauer (V-P), nitrate reduction, propionate, citric acid, 7% sodium chloride growth, and pH 5.7 growth tests. The enzyme identification of HF1 was performed according to methods outlined in Shoemaker and Brown (1978).

### 2.3 Phylogenetic tree construction of the HF1 strain

The different *Bacillus* species were used for phylogenetic analysis. The concatenated alignments protein sequences of HF1 were compared with those of various *Bacillus* species, Orthogroup and phylogenetic tree inference using the OrthoFinder (version 2.5.4) software with parameter “-s blast-M msa-T raxml-I 1.3-t 10” and HF1 genome sequence (see 2.5 for genome sequencing of HF1). OrthoFinder’s comprehensive phylogenetic analysis are able to distinguish variable sequence evolution rates from the order in which sequences diverged and hence clarify orthology and paralogy relationships.

### 2.4 Anti-fungal tests on the HF1 strain

The dural culture method was used to test the effect of the live HF1 strain and the sterile supernatant of the HF1 fermentation broth on resistance to different fungi (listed in Table 1). Briefly, a fungal cake with a diameter of 6 mm was placed in the center of the PDA medium, and 1 μL suspension of HF1 live bacteria was inoculated into the site approximately 30 mm from the center of the fungal cake. When the fungus in the control group was spread over the entire medium plate, the antagonistic effect of HF1 bacteria on the fungus was measured. The following formula was used to calculate the inhibition rate: Inhibition rate (%) = diameter of the fungus (the control group) – diameter of the fungus (the treatment group)/diameter of the fungi (the control group) × 100%. The sterile supernatant of the HF1 fermentation broth was prepared by the following procedure. HF1 was inoculated at 2% into triangular flasks containing LB medium and shaken for 48 h at 28°C and 200 rpm. The culture was then centrifuged at 10,000 rpm at 4°C for 15 min, and the resulting supernatant was collected and filtered through a 0.22 μm filter membrane to eliminate bacteria, yielding a sterile filtrate. Sterile supernatant (200 μL) was placed into a hole 30 mm from the center where the fungus cake was inoculated. The inhibition rate was calculated when the control group grew over the plate using the formula described above. The experiment was repeated 3 times.

**Table 1:** Inhibitory effects of *Bacillus subtilis* HF1 on 20 species of fungi.

Fungi	Inhibition rate (%) of live bacteria	Inhibition rate (%) of sterile supernatants
*Trichoderma viride*	76.61 ± 0.03	71.52 ± 0.45
*Botryospuaeria dothidea*	66.66 ± 1.31	67.43 ± 2.50
*Glomerella cingulat*	65.63 ± 2.29	24.73 ± 1.94
*Alternaria alternata* (Fr) Keissler	64.30 ± 3.93	54.81 ± 0.02
*Curvularia trifolii*	59.95 ± 1.46	60.66 ± 2.93
*Verticillium dahlia*	59.93 ± 3.23	32.54 ± 4.69
*Botrytis cinerea*	54.01 ± 3.58	57.12 ± 3.02
*Piriformospora indica*	52.23 ± 6.81	47.71 ± 2.55
*Magnaporthe grisea* 3–2	51.84 ± 1.47	64.68 ± 4.37
*Valsa mali*	51.68 ± 2.56	62.91 ± 1.48
*Alternaria gossypina*	51.65 ± 1.93	55.16 ± 3.45
*Alternaria alternata* f. sp. mali	48.30 ± 1.95	46.13 ± 1.94
*Corynespora cassiicola*	46.98 ± 2.09	48.26 ± 2.00
*Sclerotinia sclerotiorum*	44.17 ± 3.63	51.79 ± 1.55
*FusaHum graminearum*	43.28 ± 1.67	36.91 ± 1.70
*Fusarium oxysporum*	41.07 ± 2.36	25.00 ± 3.37
*Phyllosticta brassicea*	39.73 ± 2.04	52.20 ± 2.51
*Lasiodiplodia theobromae*	37.38 ± 4.27	50.84 ± 2.54
*Fusarium verticillioides*	34.59 ± 1.62	25.99 ± 1.69
*Pestalotiopsis microspora*	33.63 ± 1.57	38.82 ± 0.05
*Fusarium equiseti*	31.06 ± 1.68	30.26 ± 1.58

Inhibitory effects of bacterial cells and sterile supernatants of *B. subtilis* HF1 on the mycelial growth of 20 fungal species. The values are the means ± standard errors of three biological replicates.

### 2.5 Genome sequencing, assembly, and secondary metabolite prediction

The genomic DNA of strain HF1 was extracted using the M5 Bacteria Genomic DNA Kit purchased from Beijing Mei5 Biotechnology (Beijing, China). The extracted HF1 genomic DNA was sequenced and assembled *de novo* by Biomarker Technologies (Beijing, China) using a next-generation sequencing method (Nanopore sequencing). The fast5 format data were converted to fastq format after base calling by guppy 3.2.6 software, and the total dataset was obtained after further filtering the reads of linkers and low-quality and short fragments (< 2,000 bp in length). The filtered subreads were assembled using Canu v1.5/wtdbg v2.2 software. Finally, Pilon v1.22 software was used to further correct errors in the assembled genome using the second-generation data, after which the genome with a higher accuracy was obtained. The online prediction software antiSMASH5.0^1^ was used to predict and analyze the secondary metabolite synthesis gene clusters of strain HF1 to identify potential antimicrobial metabolites. The genome sequence data of HF1 (GSA: CRA018930) is available at: https://ngdc.cncb.ac.cn/gsa, and genome assembly sequence (accession number: GWHFDPU01000000) is available at: https://ngdc.cncb.ac.cn/gwh.

### 2.6 Isolation and characterization of antifungal lipopeptides

Antifungal lipopeptides of HF1 were obtained using the acid precipitation method (Smyth et al., 2016) and visualized by thin layer chromatography (TLC) (Smyth et al., 2016). In detail, HF1 was fermented for 48 h using LB medium at 28°C and 200 rpm, and the supernatant was obtained by centrifugation at 10,000 × g for 20 min. The supernatant was adjusted to pH 2.0 with 6 M HCl and placed at 4°C overnight. The next day, the supernatant was discarded after centrifugation at 10,000 × g for 15 min, after which the precipitate was retained. After adding 5 mL of methanol to dissolve the precipitate, the solution was centrifuged at 10,000 × g for 10 min, and the lipopeptides in the methanol solution were filtered through a 0.22 μm filter membrane for later use.

The HF1 lipopeptide crude extract (6 μL) was placed on a silica gel plate, followed by chromatography, and the resulting lipopeptides were mixed with 3% ninhydrinin. The antimicrobial lipopeptide components separated on the silica gel plate were scraped off, dissolved in methanol and filtered through a 0.22 μm filter membrane, and the molecular weights of the components were determined by matrix-assisted laser desorption/ionization time-of-flight mass spectrometry (MALDI-TOF-MS, UltrafleXtreme, Brucker, Germany).

The structure of the lipopeptide molecule in the fermentation broth was identified by ultrahigh-performance liquid chromatography with quadrupole time-of-flight mass spectrometry (UPLC-Q-TOF-MS, Agilent 6,530, Agilent, USA). The liquid chromatography parameters were as follows: mobile phase A was 0.1% formic acid, and mobile phase B was methanol. The elution gradient was as follows: 0.1 min, 70% B; 0.1–2.0 min, 70% B; 2.0–8.0 min, 70–100% B; 8.0–10 min, 100% B; 10.1 min, 70% B; 10.1–13 min, 70% B. The flow rate was 0.3 mL/min, and the chromatographic column used was a Waters BEH C18 (50 mm × 2.1 mm, 1.7 μm particle). For mass spectrometry, positive ion mode was used for analysis with the following MS tuning parameters: degassing speed, 500 L/h; degassing temperature, 500°C; cone gas flow, 50 L/h; source temperature, 120°C; capillary voltage and cone voltage, 3.0 kV and 30 kV, respectively. The data collection time was 0.5 s, and the mass-to-charge ratio ranged from 100 ~ 1,200 m/z. The collision voltage was set to 25 V and fine-tuned according to the actual situation.

### 2.7 Quantitative analysis of the lipopeptides

High-performance liquid chromatography (HPLC) was used to quantitatively analyze surfactin and fengycin in the fermentation broth. The columns were powered by an Agilent ZORBAX SB-C18 (3.0 × 150 mm). The ultraviolet detection wavelength was 214 nm, the column temperature was 28°C, the sample load was 20 μL, the flow rate was 1 mL/min, phase A was 0.1% trifluoroacetic acid (TFA) + water, phase B was acetonitrile +0.1% TFA. The elution gradient was as follows: 0 min, 70% A, 30% B; 5 min, 52% A, 48% B; 40 min, 44% A, 56% B; 45 min, 30% A, 70% B; 60 min, 0% A, 100% B. Surfactin standards (Sigma, ≥98.0% (HPLC)) were prepared with different concentration gradients: 0.1 g/L, 0.5 g/L, 1 g/L, 5 g/L, and 10 g/L. Fengycin standards (Sigma, ≥90.0% (HPLC)) were prepared with different concentration gradients: 0.5 g/L, 1 g/L, 1.25 g/L, and 2.5 g/L. Standard curves of surfactin and fengycin were made, and the contents of surfactin and fengycin were calculated according to the peak area that was measured with HPLC (Agress 1,100, Elite, Dalian, China).

### 2.8 Strain HF1 growth curves and fengycin and surfactin synthesis curves

*Bacillus subtilis* HF1 was cultured for 84 h in a 250 mL flask with 100 mL of LB liquid medium at 28°C at 200 rpm. The OD600 value of the HF1 culture and the fengycin and surfactin contents were recorded at different culture times, and the experiment was repeated three times.

### 2.9 Determination of the stability of antifungal substances

We detected the stability of antifungal substances by testing the activity of strain HF1 fermentation broth supernatant on a representative fungus *A. alternata* under stress treatments including high temperature, high and low pH, different ions, PEG and Tween. Strain HF1 was fermented for 36 h, the fermentation broth was centrifuged for 10 min at 10,000 g, and the supernatant was obtained. For the protease resistance test, the supernatant was treated with pepsin K (1 mg/mL), pH 7.0, at 37°C for 6 h, trypsin (1 mg/mL), pH 7.0, at 30°C for 6 h, or chymotrypsin K (1 mg/mL), pH 8.0, at 37°C for 6 h. For the thermostability test, the fermentation supernatant was treated at 37°C, 60°C, 80°C or 100°C for 30 min or 1 h. For the pH stability test, the fermentation supernatant was adjusted to pH 3, 5, 7, 8, 9, or 11 with 0.1 M NaOH or 0.1 M HCl, kept at room temperature for 24 h, and then adjusted to the original pH of 8.3. The untreated group (pH 8.3) was used as a positive control, and PBS buffer (pH 8.3) was used as a negative control. For the metal ion stability tests, 1.0 M solutions of Fe^2+^, Al^3+^, Mg^2+^, K^+^, Cu^2+^, Na^+^, Ca^2+^, Zn^2+^ and Mn^2+^ were prepared and filtered through a 0.22 μm filter. Ionic solution (40 μL) was added to 2 mL of fermentation supernatant and incubated at 28°C for 4 h. For the PEG and Tween treatment tests, 20 μL of 1 mg/mL PEG 6000, Tween 20 or Tween 80 was added to 1 mL of sterile supernatant and incubated at 28°C for 4 h. The activity of the antifungal substances treated with the factors outlined above was assessed for their inhibitory activity against *A. alternata* using the dural culture method.

### 2.10 Broiler feeding experiment with HF1 as a feed additive

White-or yellow-feathered broilers were used as experimental animals, and negative control (basal diet), antibiotic positive control (basal diet with 15 mg/kg Virginiamycin), *B. subtilis* positive control (purchased from the market) and HF1 experimental groups were established with 4 columns in each group, with 10 chickens in each column, half male and half female.

The broilers in the negative control group and antibiotic positive control group (supplemented with 15 mg/kg Virginiamycin) were fed a basal diet, and the positive control group (purchased from the market) was fed a basal diet supplemented with 2 × 10^10^ cfu/kg *B. subtilis*. The HF1 experimental group was fed a basal diet, and the amount of *B. subtilis* HF1 fermentation solution added was approximately 4 × 10^9^ cfu/mL; the feeding dose, which is the ratio of the additive HF1 to drinking water, ranged from 0.05–0.8%. The white-feathered broilers were fed for 42 days and weighed, and the yellow-feathered broilers were fed for 63 days and weighed.

## 3 Results

### 3.1 Isolation and identification of *Bacillus subtilis* HF1

**Figure 1:**
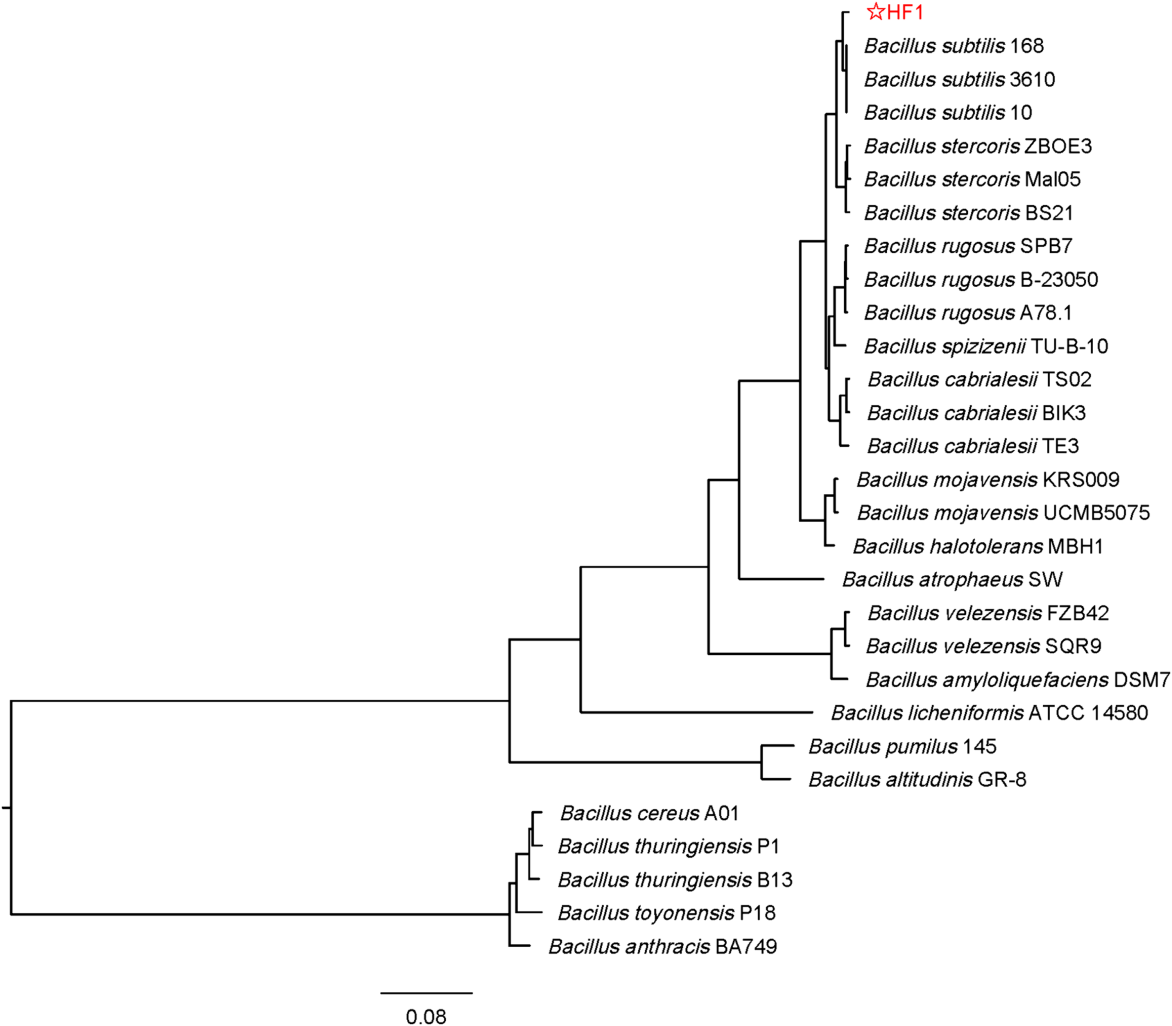
Phylogenetic tree of strain HF1. The tree is inferred by OrthoFinder using STAG algorithm and rooted by STRIDE algorithm. The species tree based on concatenated alignments of protein sequences and Orthogroup inference using the original OrthoFinder algorithm and genome sequence. The phylogenetic tree is finally drawn and marked by FigTree software and the genetic distance label is 0.08. The four closely related species of *B. subtilis* such as *B. rugosus*, *B. cabrialesii*, *B. stercoris*, and *B. spizizenii* were included in the tree. The original data were downloaded from: https://www.ncbi.nlm.nih.gov.

HF1, a bacterial strain with strong antifungal ability, was isolated from the cucumber rhizosphere soil. Based on the physiological and biochemical characterization of HF1, the strain could utilize glucose, maltose, sucrose, D-fructose, D-arabinose, D-mannitol, D-xylose, and sorbitol (Supplementary Table S1). The results of the enzyme identification experiments showed that HF1 could produce a variety of enzymes, such as amylase, cellulase, protease and *β*-1,3-glucanase (Supplementary Figure S1). The species tree of strain HF1 showed that the highest genome sequence similarity was present between HF1 and *B. subtilis* (Figure 1). The stain HF1 was eventually identified as *Bacillus subtilis* HF1.

### 3.2 *Bacillus subtilis* HF1 has broad-spectrum antifungal activity

The antifungal activity of the bacterial cells and sterile supernatants of *B. subtilis* HF1 fermentation broth was tested, and the results showed that HF1 had a significant inhibitory effect on 20 tested species of filamentous fungi (Table 1; Supplementary Figure S2), with a 24.73–76.61% inhibition rate. HF1 had greater inhibitory effects on *Trichoderma viride*, *Botryospuaeria dothidea* and *Glomerella cingulat* (65.63–76.61%, respectively). The inhibition rates of sterile supernatants of HF1 fermentation broth against *Glomerella cingulat*, *Fusarium oxysporum* and *Fusarium verticillioides* were approximately 25%.

### 3.3 Genetic basis of the antifungal activity of *Bacillus subtilis* HF1

**Figure 2:**
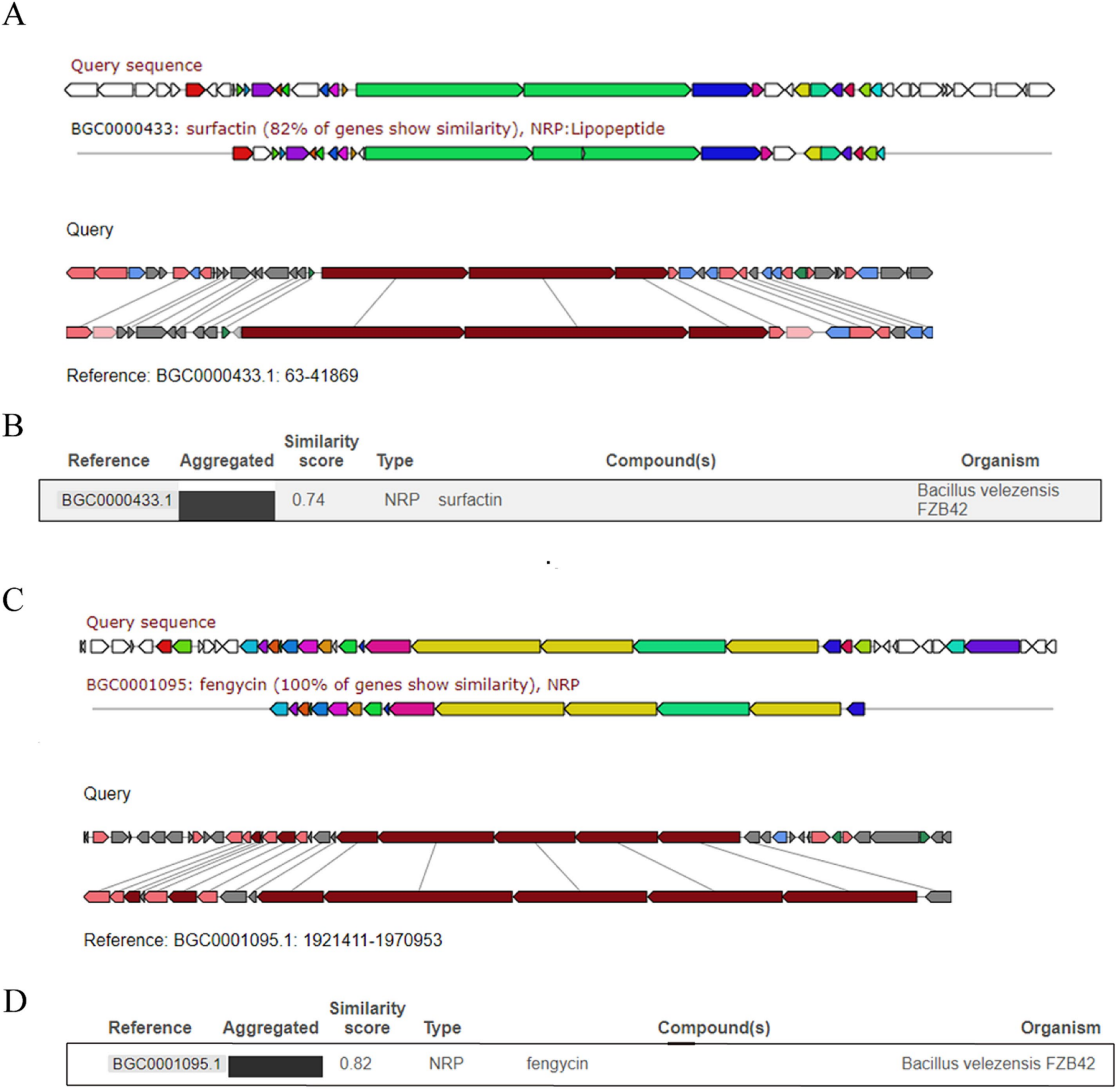
Comparisons of surfactin and fengycin gene clusters between *B. subtilis* HF1 and *B. velezensis* FZB42. Comparison and analysis of the similarities of the surfactin gene cluster **(A,B)** and fengycin gene cluster **(C,D)** between *B. subtilis* HF1 and *B. velezensis* FZB42 by region-to-region type.

To clarify the antimicrobial effects and possible underlying mechanisms of *B. subtilis* HF1 against filamentous fungi, the whole genome of HF1 was sequenced and assembled. The size of the whole genome of HF1 was 4,079,604 bp, and the GC content was 43.87%. Compared with those of *B. subtilis* 168 and *Bacillus velezensis* FZB42, the basic genomic characteristics of HF1 and *B. subtilis* 168 were the most similar (Supplementary Table S2). According to the genome sequence, HF1 contained six potential antimicrobial secondary metabolite gene clusters synthesizing surfactin, fengycin, bacillibactin, bacylisin, bacillaene and subtilosin A that had a total size of approximately 164.2 kb, accounting for approximately 4% of the whole-genome sequence. Surfactin, fengycin, bacillibactin and bacylisin are synthesized via the nonribosome pathway (NRPS), bacillaene is synthesized via the polyketide synthase pathway (PKS), and subtilosin A is synthesized via the ribosome synthesis pathway (RPS). Compared with *B. velezensis* FZB42, which is an excellent strain that produces multiple secondary metabolites (Koumoutsi et al., 2004), *B. subtilis* HF1 has fewer gene clusters for antimicrobial metabolites and has no iturin, macrolactin or difficidine gene clusters (Table 2). HF1 has the same antifungal gene clusters as the model strain *B. subtilis* 168. Sequence alignment analysis revealed that 82% of the surfactin genes in *B. subtilis* HF1 and *B. velezensis* FZB42 were similar, and regional cluster analysis revealed that the homology score reached 0.74 (Figures 2A,B); the similarity of the fengycin gene cluster of *B. subtilis* HF1 and *B. velezensis* FZB42 was 100%, and the regional cluster analysis revealed that the homology score reached 0.82 (Figures 2C,D).

**Table 2:** Prediction and comparison of antimicrobial metabolite synthesis in *B. subtilis* HF1.

Compounds	Synthetase	Gene clusters	Activity	*B. subtilis* 168	*B. velezensis* FZB42	*B. subtilis* HF1
Fengycin	NRPS	*fenABCDE*	Antifungal	√	√	√
Surfactin	NRPS	*srfABCD*	Antibacterial, antifungal	√	√	√
Iturin	NRPS	*bmyDABC*	Antifungal	×	√	×
Bacillibactin	NRPS	*dhbABCDEF*	Iron ion accumulation	√	√	√
Bacillaene	PKS	*baeBCDE, acpK, baeGHIJLMNRS*	Antibacterial	√	√	√
Subtilosin A	RPS	*sboA, ywiA, ywhMNOPQR*	Antibacterial	√	×	√
Macrolactin	PKS	*mlnABCDEFGHI*	Anti-inflammatory,	×	√	×
			anticancer,			
			antibacterial			
Difficidine	PKS	*dfnAYXBCDEFGHIJKLM*	Antibacterial, antifungal	×	√	×
Bacylisin	NRPS	*BacABCDEywfAG*	Antifungal	√	√	√

The NRPS, PKS, and RPS gene clusters are involved in the synthesis of secondary metabolites in *B. subtilis* HF1, *B. subtilis* 168 and *B. velezensis* FZB42. In *B. subtilis* 168, although there is a surfactin gene cluster, it does not produce surfactin.

### 3.4 Isolation and identification of antifungal lipopeptides

**Figure 3:**
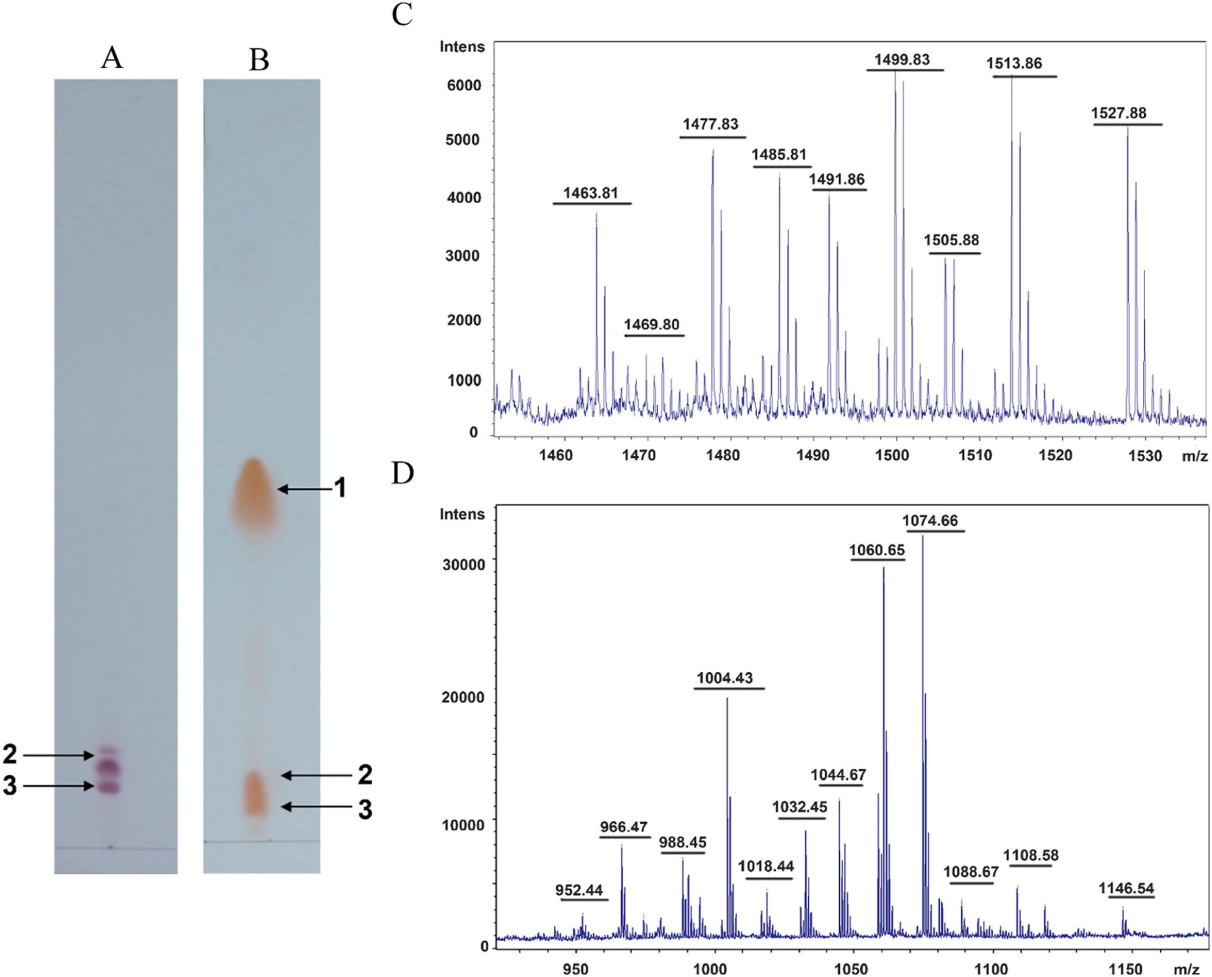
Isolation and identification of antifungal lipopeptides. **(A)** Thin layer chromatography detection of the nonhydrolysed sample. **(B)** Thin layer chromatography detection of the acid-hydrolysed sample. 1, 2, and 3 represent the antimicrobial compounds produced from *B. subtilis* HF1. **(C,D)** MALDI-TOF-MS of samples a and b. Sample a is composed of a mix of components 2 and 3 isolated from thin layer chromatography, and sample b is composed of component 1 isolated from thin layer chromatography.

To determine the composition of the antimicrobial substances of *B. subtilis* HF1, the crude extract of the sterile supernatant of HF1 fermentation broth was analyzed by thin layer chromatography (TLC). We found that the acid-hydrolysed and non-acid-hydrolysed components had the same orange–yellow spots at positions 2 and 3 (Figures 3A,B), indicating that these two components contained free amino acids. Orange–yellow spots appeared at position 1 after acid hydrolysis, which indicated that the cyclic peptide structure was opened after acid hydrolysis and that the free amino group was exposed. It was postulated that there was a closed peptide bond in the HF1 antibacterial crude extract at spot 1 (Figures 3A,B). The spot 2 and 3 components separated by thin layer chromatography were mixed as sample a, and the spot 1 component was used as sample b. The molecular weights of samples a and b were detected by matrix-assisted laser desorption/ionization time-of-flight mass spectrometry (MALDI-TOF-MS). The molecular weight of sample a was between 1,400–1,550 Da, which was similar to that of fengycin lipopeptides. The molecular weight of sample b was in the range of 900–1,200 Da, which was similar to that of surfactin lipopeptides (Figures 3C,D).

### 3.5 The types and homologs of lipopeptides in *Bacillus subtilis* HF1

**Figure 4:**
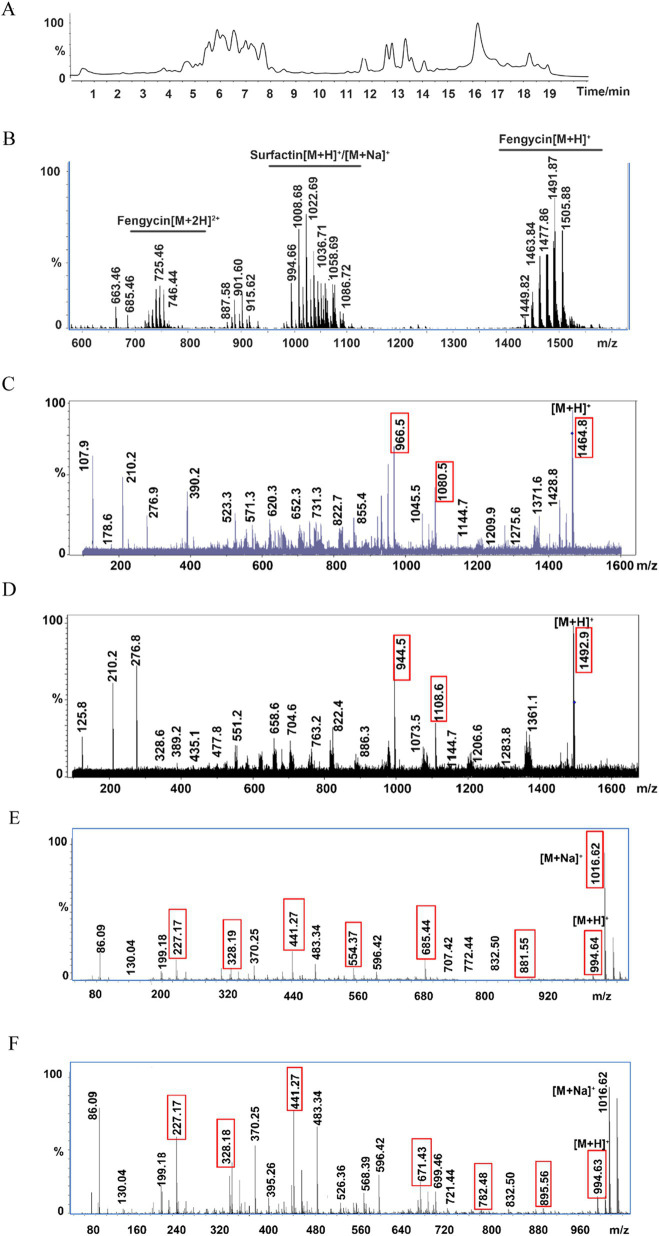
Ultrahigh-performance liquid chromatography with quadrupole time-of-flight mass spectrometry (UPLC-Q-TOF-MS) for lipopeptide compound analysis. **(A)** Total ion chromatography of the lipopeptides by LC–MS. **(B)** ESI-MS spectrum of the lipopeptides. **(C)** MS/MS spectrum of m/z 732.41 ([M + 2H]^2+^); the two red marked fingerprint product ions 1,080 and 966 suggested that m/z 732.41 belongs to fengycin A. **(D)** MS/MS spectrum of m/z 746.43 ([M + 2H]^2+^); the two red marked fingerprint product ions 1,108 and 994 suggested that m/z 746.43 belongs to fengycin B. **(E)** MS/MS spectrum of m/z 994.64 (surfactin A); the red marked fingerprint product ions are daughters of m/z 994.64. **(F)** MS/MS spectrum of m/z 994.63 (surfactin B); the red marked fingerprint product ions are daughters of m/z 994.63.

The types of lipopeptide compounds were identified and analyzed by ultrahigh-performance liquid chromatography with quadrupole time-of-flight mass spectrometry (UPLC-Q-TOF-MS), and the total ion chromatography (TIC) results of the samples exhibited two signal peaks (Figure 4A), which were concentrated at 4.5–8.5 min and 11–15 min, respectively. The mass–charge ratio of 4.5–8.5 min is in the range of 1,400-1,500, and a double-charged ion [M + 2H]^2+^ is formed, which is the signal peak of fengycin homologs and isomers. The mass–charge ratio for 11–15 min ranged from 900 to 1,200, forming two kinds of charged ions, [M + H]^+^ and [M + Na]^+^, which are the signal peaks of surfactin homologs and isomers (Figure 4B). The ion peaks at m/z 1,080 and 966 in the secondary mass spectrometry are characteristic of fengycin A (Bie et al., 2009); the m/z after the loss of fatty acid and adjacent Glu from the N-terminus of fengycin A was 1,080, and the m/z after the loss of fatty acid and adjacent Glu-Orn was 966. The ion peaks at m/z 1,108 and 994 in the secondary mass spectrometry are characteristic of fengycin B (Bie et al., 2009). After the loss of fatty acid and adjacent Glu from the N-terminus, the m/z was 1,108. The m/z of the loss of fatty acids and adjacent Glu-Orn was 994 (Wang et al., 2024; Supplementary Figure S3). The double proton ion peak [M + 2H]^2+^ of *B. subtilis* HF1 was selected as the leading ion for MS/MS analysis. The results showed that there were characteristic ion peaks with m/z of 1,080 and 966 in the fragment ion of secondary mass spectrometry, and it was identified as fengycin A (Figure 4C). The ion peaks with m/z of 1,108 and 994 were identified as fengycin B (Figure 4D). Based on the ESI-MS/MS mass spectrometry data, a total of 13 homolog compounds of fengycin were identified in this study, as shown in Table 3, including C_13_-C_19_ fengycin A and C_15_-C_17_ fengycin B, as well as C_17_ fengycin A, C_15_ fengycin B and C_16_ fengycin B containing an unsaturated double bond.

**Table 3:** Molecular identification of fengycins in *B. subtilis* HF1.

Leading ion m/z [M + 2H]^2+^	Leading ion m/z [M + H] ^+^	Molecular weight (Da)	Characteristic ions (m/z)	Fengycin homologs
711.38	1,422	1,421	1,080 and 966	C_13_ fengycin A
718.38	1,436	1,435	1,080 and 966	C_14_ fengycin A
725.39	1,450	1,449	1,080 and 966	C_15_ fengycin A
732.41	1,464	1,463	1,080 and 966	C_16_ fengycin A
739.41	1,478	1,477	1,080 and 966	C_17_ fengycin A
738.41	1,476	1,475	1,080 and 966	C_17*_ fengycinA
746.43	1,492	1,491	1,080 and 966	C_18_ fengycin A
753.42	1,506	1,505	1,080 and 966	C_19_ fengycin A
746.43	1,492	1,491	1,108 and 994	C_16_ fengycin B
739.41	1,478	1,477	1,108 and 994	C_15_ fengycin B
738.42	1,476	1,475	1,108 and 994	C_15*_ fengycin B
745.43	1,490	1,489	1,108 and 994	C_16*_ fengycin B
753.42	1,506	1,505	1,108 and 994	C_17_ fengycin B

Identification of fengycin homologs in the lipopeptide extract of *B. subtilis* HF1. *Refers to one double bond in the fatty acid chain.

Surfactin, an excellent biosurfactant, can be found in two main types: surfactin A in which the 7th position of the cyclic lipopeptide is Leu and surfactin B, in which the amino acid at the 7th position of the cyclic lipopeptide is Val (Supplementary Figure S4). Among the secondary mass spectrometry fragments of surfactin A, the most abundant m/z 685 represents the product of glutamate-leucine and fatty acid-leucine cleavage and the hexapeptide residue Leu_2_-Leu_3_-Val_4_-Asp_5_-Leu_6_-Leu_7_; the other more abundant ion fragment m/z 441 represents the Leu_2_-Leu_3_-Val_4_-Asp_5_ tetrapeptide residue, and the m/z 671 of the surfactin B secondary mass spectrometry ion fragment represents the Leu_2_-Leu_3_-Val_4_-Asp_5_-Leu_6_-Val_7_ hexapeptide residue, while the same relatively abundant m/z 441 represents the Leu_2_-Leu_3_-Val_4_-Asp_5_ tetrapeptide residue (Korenblum et al., 2012). The *B. subtilis* HF1 m/z 994.64 ion was selected for ESI-MS/MS analysis, and among the secondary mass spectrometry fragments, m/z 685 (Leu_2_-Leu_3_-Val_4_-Asp_5_-Leu_6_-Leu_7_) and another more abundant ion fragment mass peak, m/z 441 (Leu_2_-Leu_3_-Val_4_-Asp_5_), followed by m/z 881 (FA-Glu_1_-Leu_2_-Leu_3_-Val_4_-Asp_5_-Leu_6_), m/z 328 (FA-Glu_1_) and m/z 227 (Leu_6_-Leu_7_), were identified as belonging to surfactin A (Figure 4E); the same m/z 994.63 ion in *B. subtilis* HF1 had the highest relative abundance of m/z 671 (Leu_2_-Leu_3_-Val_4_-Asp_5_-Leu_6_-Val_7_) and another more abundant ion fragment mass peak, m/z 441 (Leu_2_-Leu_3_-Val_4_-Asp_5_), followed by m/z 895 (FA-Glu_1_-Leu_2_-Leu_3_-Val_4_-Asp_5_-Leu_6_), m/z 782 (FA-Glu_1_-Leu_2_-Leu_3_-Val_4_-Asp_5_), m/z 568 (FA-Glu_1_-Leu_2_-Leu_3_), m/z 328 (FA-Glu_1_) and m/z 227 (Leu_6_-Leu_7_), were identified as C_13_ surfactin B (Figure 4F). In this study, 11 kinds of surfactin homologs were identified, namely, C_11_-C_17_ surfactin A and C_13_-C_16_ surfactin B, as shown in Table 4. Among them, C_11_ and C_17_ surfactin A were rare surfactants and may have special functions.

**Table 4:** Molecular identification of surfactins in strain HF1.

Leading ion m/z [M + Na]^+^	Leading ion m/z [M + H] ^+^	Molecular weight (Da)	Surfactin homologs
1002.6098	980.6286	979.6	C_11_ surfactin A
1016.6254	994.6597	993.6	C_13_ surfactin B
1016.6254	994.6480	993.6	C_12_ surfactin A
1030.6411	944.6480	933.6	C_13_ surfactin A
1030.6415	1008.6586	1007.6	C_14_ surfactin B
1044.6575	1022.6752	1021.6	C_14_ surfactin A
1044.6568	1022.6739	1021.6	C_15_ surfactin B
1058.6735	1036.6910	1035.6	C_15_ surfactin A
1058.6735	1036.6910	1035.6	C_16_ surfactin B
1072.6884	1050.7067	1049.7	C_16_ surfactin A
1086.7037	1064.7217	1063.7	C_17_ surfactin A

Identification of surfactin homologs in the lipopeptide extract of *B. subtilis* HF1.

### 3.6 Quantitative analysis of surfactin and fengycin in *Bacillus subtilis* HF1

**Figure 5:**
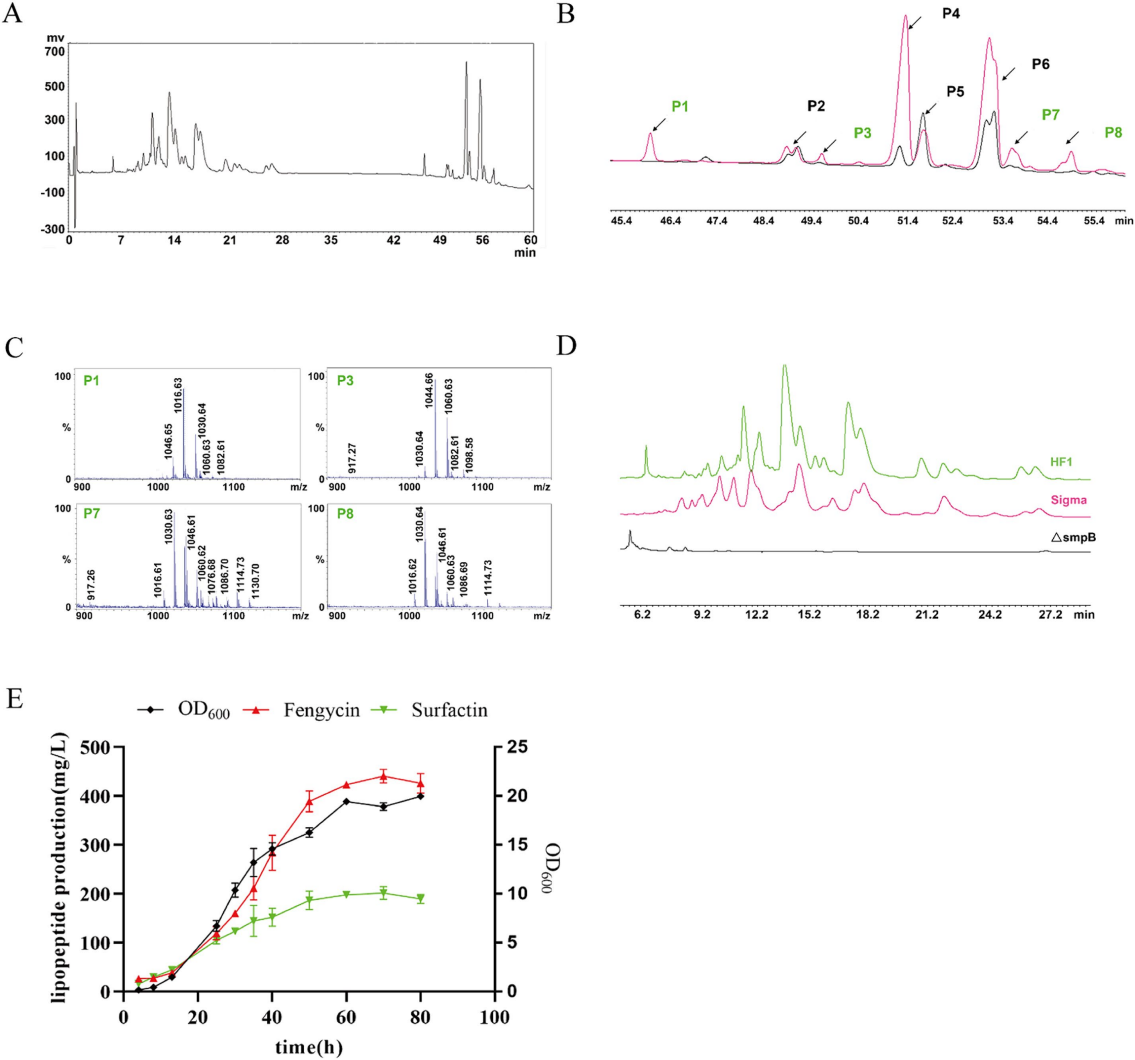
Surfactin and fengycin crude extracts were quantitatively analyzed by HPLC. **(A)** HPLC chromatogram of the crude methanolic extract of *B. subtilis* HF1. **(B)** HPLC chromatograms of surfactin standards (Sigma) and surfactins in the crude methanolic extract of *B. subtilis* HF1. The signal peaks P2, P4, P5, and P6 marked in black represent the overlapping peaks of surfactins in the crude methanolic extract from *B. subtilis* HF1 and surfactin standards (sigma), and the signal peaks P1, P3, P7 and P8 marked in green represent the unique peaks of surfactins in the crude methanolic extract from *B. subtilis* HF1. **(C)** MALDI-TOF-MS mass spectra of signal peaks P1, P3, P7, and P8. **(D)** HPLC chromatograms of fengycin standards (Sigma) and fengycin in the crude methanolic extract of *B. subtilis* HF1. **(E)** Growth curve and surfactin and fengycin production curve of *B. subtilis* HF1.

The crude extracts extracted by methanol were quantified by HPLC using the external standard curve method, and the optimized HPLC separation procedure for the crude antimicrobial extracts was used (Supplementary Table S3). The methanol crude extract of *B. subtilis* HF1 was separated by HPLC to obtain two signal peaks at 7.4–28 min and 45–55 min (Figure 5A). The signal peaks P2, P4, P5 and P6 of the surfactin standard and the crude extract of *B. subtilis* HF1 overlapped within 45.5–55 min, the signal peaks P1, P3, P7 and P8 unique to strain HF1 were collected separately, and the signal peaks P1, P3, P7 and P8 were detected by MALDI-TOF-MS mass spectrometry. The results showed that the signal peak of the crude extract of *B. subtilis* HF1 fermentation broth at 45.5–55 min was attributed to surfactin (Figures 5B,C). Most of the signal peaks of the fengycin standard and the crude extract of *B. subtilis* HF1 overlapped at 7.6–26.7 min. The main peaks were detected by UPLC-Q-TOF-MS mass spectrometry, and the peaks at 7.6–26.7 min were identified as fengycin (Figure 5D). The standard sample curves of surfactin and fengycin (Sigma) were drawn based on the peak area detected at 214 nm. The lipopeptide content in the *B. subtilis* HF1 crude extract was calculated based on the peak area, and the contents of surfactin and fengycin reached 211.41 mg/L and 457.061 mg/L, respectively, after shaking for 48 h at 28°C and 200 rpm. By measuring the surfactin and fengycin contents and the growth of strain HF1, it was found that the synthesis curves of surfactin and fengycin were consistent with the growth curves of *B. subtilis* HF1. The lipopeptide content rapidly increased in the logarithmic phase and reached the highest level in the stable phase (Figure 5E).

### 3.7 The antifungal lipopeptides have excellent stability

**Figure 6:**
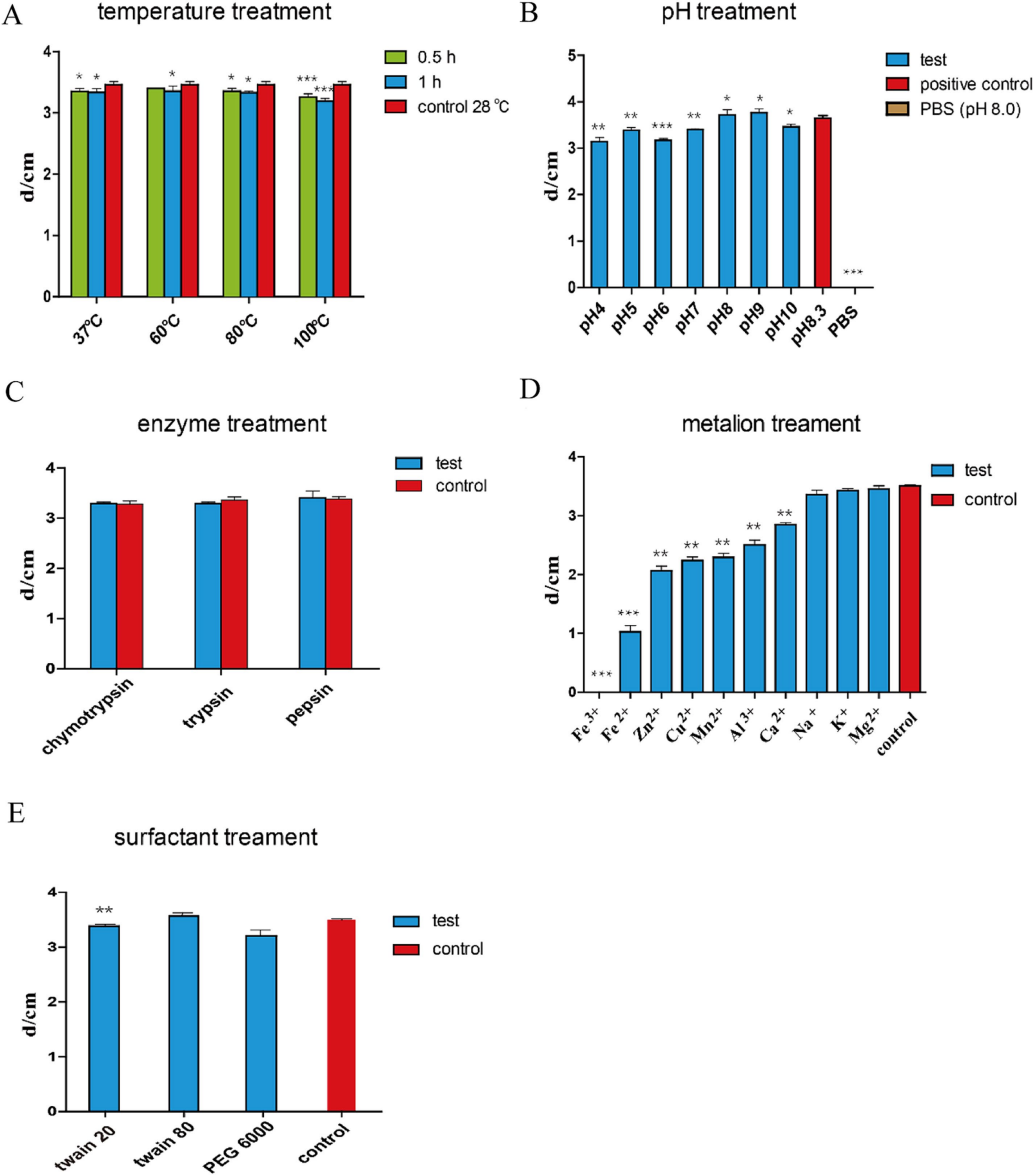
Stability tests of the antifungal active substances of *B. subtilis* HF1. The supernatant of the fermentation broth of *B. subtilis* HF1 was subjected to different temperatures [**(A)** treatment for 0.5, and 1 h], pH [**(B)** 25°C treatment for 24 h], enzyme [**(C)** concentration 1 mg/mL, treatment for 6 h], metal ions [**(D)** concentration 1 M, 25°C treatment for 4 h], and surfactant [**(E)** 1 g/mL, 25°C treatment for 4 h]. After treatment, the antibacterial activity was measured with *A. alternata* as the indicator fungus, and the diameter of the inhibition zone was determined by the dural culture method. The control group was the untreated fermented supernatant. Each experiment was repeated three times. *** means: *p* < 0.001, ** means: *p* < 0.01, * means: *p* < 0.05.

In addition to the advantages of the wide antimicrobial spectrum of lipopeptides, the stability of the peptides is also very important and is highly important for the further application of *B. subtilis* HF1. To further investigate the properties of lipopeptides, we determined the protease sensitivity, thermal stability, acid–base stability, metal ion stability, and surfactant stability of the antimicrobial peptides in the fermentation supernatant of *B. subtilis* HF1. The results showed that antimicrobial peptides from HF1 were insensitive to proteases (Figure 6C), were acid-and alkali-resistant within a certain range (Figure 6B) and had excellent thermal stability (Figure 6A) and surfactant stability (Figure 6E), but they were sensitive to several metal ions, such as Fe^3+^, Fe^2+^, Zn^2+^, Cu^2+^, Mn^2+^, Al^3+^ and Ca^2+^ (Figure 6D). These results indicate that the effective antimicrobial substances of HF1 could not only withstand high temperatures but also maintain activity under certain acid–base conditions and were not easily degraded by enzymes; thus, HF1 has the potential to be used in the field of animal feed additives and plant disease control.

### 3.8 The probiotic *Bacillus subtilis* HF1 improves the growth performance of broilers

**Figure 7:**
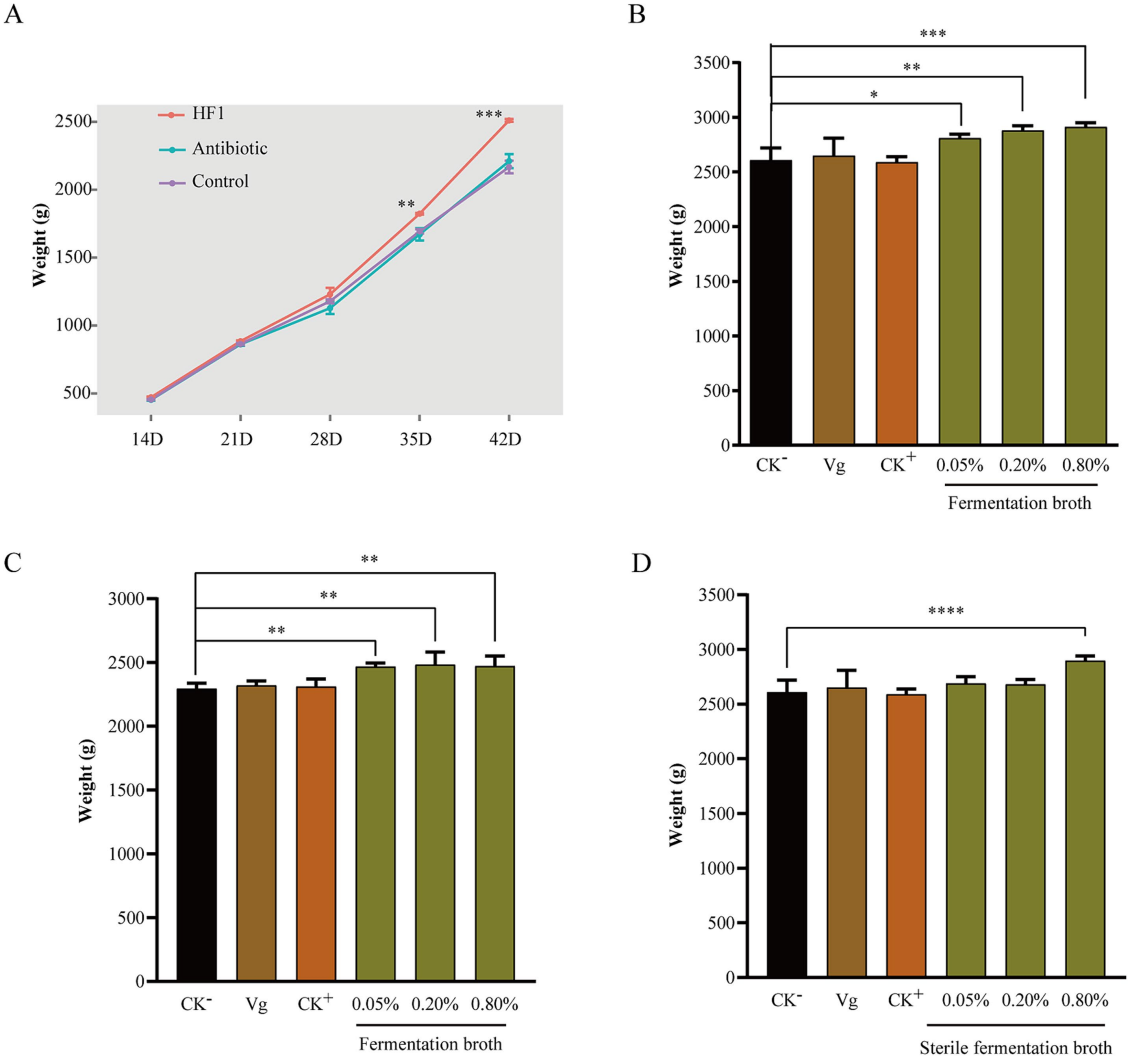
**(A)** Changes in the body weight of white-feathered broilers during 14 days–42 days of feeding under different feeding conditions. The control group was supplemented with only a basal diet, the antibiotic group was supplemented with 15 mg/kg Virginiamycin in the basal diet, and the HF1 group was supplemented with 1‰ *B. subtilis* fermentation broth in the drinking water. **(B)** The body weight of white-feathered broilers on the 42nd day after feeding with different doses of *B. subtilis* HF1 as a feed additive. CK^−^, basal diet; Vg, basal diet with 15 mg/kg Virginiamycin; CK^+^, basal diet with 1‰ *B. subtilis* microecological agent (purchased from the market). **(C)** The body weight of yellow-feathered broilers on the 63rd day after the administration of different doses of the *B. subtilis* HF1 feed additive. CK^−^, basal diet; Vg, basal diet with 15 mg/kg Virginiamycin; CK^+^, basal diet with 1‰ *B. subtilis* microecological agent (purchased from the market). **(D)** The body weight of white-feathered broilers on the 42nd day after feeding with different doses of *B. subtilis* HF1 sterile fermentation broth as a feed additive. CK^−^, basal diet; Vg, basal diet with 15 mg/kg Virginiamycin; CK^+^, basal diet with 1‰ *B. subtilis* microecological agent (purchased from the market). Each experiment was repeated four times. *** indicates *p* < 0.001, ** indicates *p* < 0.01, and * indicates *p* < 0.05.

*Bacillus subtilis* HF1 can secrete a variety of enzymes, which may promote the digestion and absorption of nutrients in broilers. To further explore the application potential of *B. subtilis* HF1, we prepared *B. subtilis* HF1 fermentation broth with and without live cells that were used as a feed additive and added to the drinking water of white-and yellow-feathered broilers and found that HF1 could significantly improve the growth performance of broilers. For white-feathered broilers, compared with those in the negative control group, there was a significant increase in body weights on the 35th day, and this increase was more significant on the 42nd day, with a percentage increase of 15.84% (Figure 7A). For yellow-feathered broilers, compared with those in the negative control group, there was a significant increase of 8.21% in body weights on the 63rd day (Figure 7C). Further experiments proved that the effect of HF1 feed additives on the weight gain of white-feathered broilers was dependent on the dose used (Figure 7B). With the increase in dose (from 0.05–0.8%) of the HF1 feed additive, the body weight gain of white-feathered broilers on the 42nd day increased from 7.74–11.70% (Figure 7B). The HF1 feed additive significantly increased the weight of broilers at a dose of 0.05%. Furthermore, we also demonstrated the benefit of HF1 sterile fermentation broth (containing antimicrobial lipopeptides) on weight gain in white-feathered broilers, whose weights increased by 11.30% at a dose of 0.8% of the additive (Figure 7D), and the minimum effective dose of sterile fermentation broth was greater than that of fermentation broth containing live cells.

## 4 Discussion

*Bacillus subtilis* is recognized as a probiotic and is widely used in agricultural production. However, the functions of different *Bacillus* spp. strains are quite diverse; some strains are used for the prevention and control of plant diseases, while others are used to promote the growth of farmed animals. The isolation of useful and powerful strains has always been a goal in this field. In this study, a new strain of *B. subtilis*, HF1, was identified and shown to have the potential to be developed as a biocontrol agent with broad-spectrum antifungal properties and as an effective feed additive to significantly promote the growth of broilers.

Our study showed that HF1 could inhibit the growth of 20 species of tested fungi and had the greatest effect on the plant pathogens *B. dothidea*, *G. cingulat* and *A. alternata*, for which the inhibition rate reached 64.30–66.66%. *B. dothidea* is the fungus that causes apple ring rot, one of the three major diseases of apples, and infection with this fungus causes the rot of apples and severe yield loss (Feng et al., 2023; Xin et al., 2023). *G. cingulat* is one of the most destructive fungal diseases of apple and seriously affects the quality and yield of apples (Liu et al., 2023). Tobacco brown spot is caused by *A. alternata* and affects the quality of tobacco (Yang et al., 2018; Cai et al., 2023). Currently, chemical control is still the main method used to control these diseases. Due to its significantly effective inhibition of the pathogens *B. dothidea*, *G. cingulat* and *A. alternata, B. subtilis* HF1 provides a new option for the prevention and control of these plant fungal diseases.

The basis of the antifungal activity of *B. subtilis* is the production of secondary metabolites, of which lipopeptides are important antifungal metabolites (Zhao et al., 2019; Zhao et al., 2017; Desmyttere et al., 2019; Kang et al., 2020). In the lipopeptide family, surfactin and fengycin are two important lipopeptides, and the types and contents of surfactin and fengycin determine their antifungal ability (Tao et al., 2011; Pueyo et al., 2009). In general, surfactin contains 13–15 carbon atoms, fengycin contains 14–17 carbon atoms, and the homologs produced by different strains vary greatly (Pecci et al., 2010). For example, *B. subtilis* LSFM-05 produces C_14_-C_17_ fengycin A and C_15_-C_17_ fengycin B (de Faria et al., 2011). *B. subtilis* B9-5 produces C_16_-C_19_ fengycin A and C_14_-C_16_ fengycin B (DeFilippi et al., 2018). Moreover, the functions of different homologs of fengycin are also quite different; for example, the antifungal activity of fengycin increases with the elongation of the fatty acyl chain (Deleu et al., 2008; Patel et al., 2011). In this study, we found that there were two main antifungal active substances in the HF1 fermentation broth, fengycin and surfactin; the HF1 fengycin contained C_13_-C_19_ fengycin A and C_15_-C_17_ fengycin B, with a total of 13 types and homologs, and the HF1 surfactin contained C_11_-C_17_ surfactin A and C_13_-C_16_ surfactin B, with a total of 11 types and homologs. The results showed that HF1 could be a strain that potentially produced the richest types and homologs of surfactin and fengycin to date. In addition, for the identified lipopeptides C_13_ fengycin A, C_11_ surfactin A and C_17_ surfactin A, which may have new functions and warrant further investigation, were reported for the first time in this study.

The antifungal activity was affected not only by the lipopeptide compositions but also by the content. The yield and productivity of lipopeptides can be significantly increased by improving the medium and process conditions. The carbon source, nitrogen source and the ratio between them as well as trace elements in the medium have a significant effect on the production of lipopeptides (Wang et al., 2024; Fifani et al., 2022; Chen et al., 2022). The production of lipopeptides can also be increased by adding exogenous amino acids (Zhou et al., 2019). High oxygen transfer conditions can promote the production of surfactin, and middle or low oxygen transfer conditions facilitate the fengycin yield (Rangarajan et al., 2015; de Paula Vieira de Castro et al., 2022). In this study, the contents of surfactin and fengycin reached 211.41 mg/L and 457.061 mg/L, respectively, after shaking for 48 h at 28°C and 200 rpm. We will improve the medium and culture condition to increase the yield of lipopeptides for increasing the antifungal activity of HF1 in future work.

*Bacillus subtilis* can promote the health and growth of farmed animals by improving the digestion and absorption of nutrients and the gut microbiota (Ruiz Sella et al., 2021; Kaewtapee et al., 2017; Mingmongkolchai and Panbangred, 2018; Grant A. et al., 2018). In this study, we found that HF1 fermentation broth with live cells could significantly promote the growth of white-feathered broilers and yellow-feathered broilers, with an increase in body weight of up to 15.84% (Figure 7A), which greatly benefits farmers. Furthermore, we found that the sterile fermentation broth of HF1 could also promote the growth of broilers, with an increase in body weight of up to 11.13% at 0.8% additive dose of HF1 cell-free fermentation broth, indicating that in addition to live microbial agents, the fermentation metabolites of the strain also played an important role in promoting the growth of broilers, which has also been reported in previous studies (Elhady et al., 2022; Chen and Yu, 2023). The results of the experiment to identify degradation enzymes showed that HF1 could secrete amylase, protease, cellulase and *β*-1,3-glucanase (Supplementary Figure S1), and these enzymes could promote the digestion and absorption of nutrients in broilers and might play an important role in improving metabolism and immune regulation. Recent work has shown that fengycin, which is secreted by *B. subtilis*, acts as a quorum-sensing inhibitor to affect intestinal *Staphylococcus aureus* colonization (Chung and Raffatellu, 2019); fengycin also interferes with the adhesion and biofilm formation of *S. aureus* and *Escherichia coli* (Rivardo et al., 2009), suggesting that fengycin can aid in the colonization of intestinal probiotics. The functions of the lipopeptides secreted by HF1 in promoting the growth of broilers deserve further investigation.

To further elucidate the function of HF1, we assembled the HF1 whole genome *de novo*, which revealed a genome size of 4,079,604 bp, a GC content of 43.87%, and a capacity to encode a total of 4,028 genes, which is slightly smaller than that of the model strain *B. subtilis* 168. The HF1 genome contains six potential antimicrobial metabolite gene clusters, with a total size of approximately 164.2 kb, accounting for approximately 4% of the whole-genome sequence. HF1 has an abundant number of genes for synthesizing secondary metabolites (Table 2), and can produce the richest lipopeptide types and homologs of surfactin and fengycin observed to date (Tables 3, 4). The genome data of HF1 provides a genetic basis for HF1 to have functions in both inhibiting plant fungi and promoting the growth of broilers.

In conclusion, we isolated a new strain of *B. subtilis,* HF1, which has broad-spectrum strong antifungal properties and has the potential to be developed as an effective biocontrol agent for plant disease; this strain can significantly promote the growth of white and yellow-feathered broilers and has the potential to be developed as an effective feed additive. HF1 can secrete surfactin and fengycin and can produce the richest types and homologs of these two lipopeptides observed to date, among which the C_11_ surfactin A, C_17_ surfactin A and C_13_ fengycin A homologs are reported for the first time. In this study, the whole-genome sequence of HF1 was assembled, which laid a foundation for in-depth analysis of HF1 metabolites. The antifungal mechanism and animal growth promotion mechanism of HF1 must be further studied.

## Data Availability

The original contributions presented in the study are included in the article/Supplementary material, further inquiries can be directed to the corresponding authors.

## Footnotes

^1^http://antismash.secondarymetabolites.org/#!/start
