# Supplementary material for: Isolation of a novel Bacillus subtilis HF1 strain that is rich in lipopeptide homologs and has strong effects on the resistance of plant fungi and growth improvement of broilers
Source: Front Microbiol. 2024 Oct 1;15:1433598. doi: 10.3389/fmicb.2024.1433598 (PMC11474111; doi:10.3389/fmicb.2024.1433598)
Supplement: Supplementary file 1 [file Data_Sheet_1.docx]

***Supplementary material***

1. **Supplementary Figures and Tables**

**1.1** **Supplementary Figures**


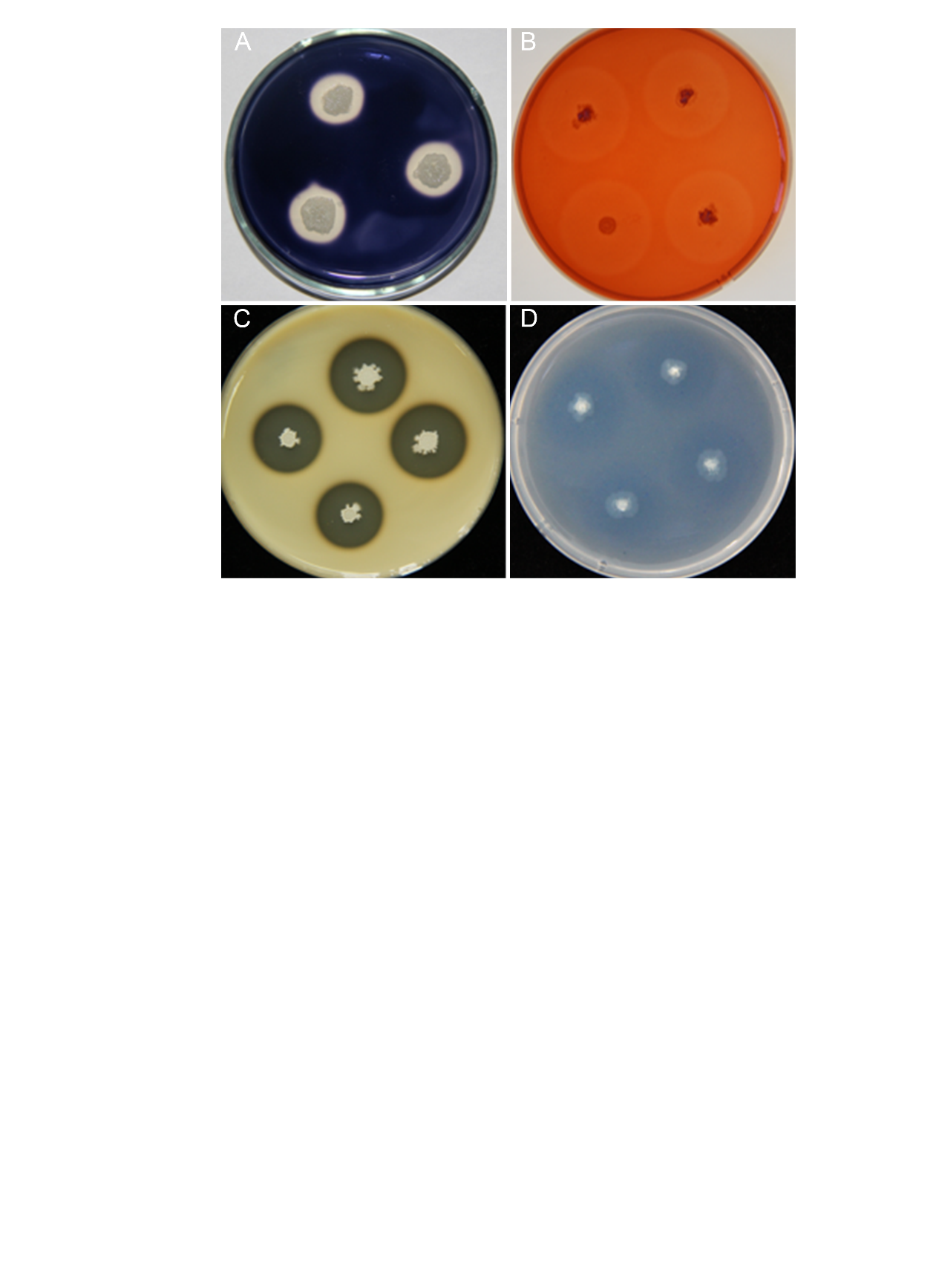


**Supplementary Figure 1.** The identification of several enzymes in strain HF1. The identification of amylase (**A**), cellulase (**B**), proteinase (**C**), and β-1,3-glucanase (**D**).


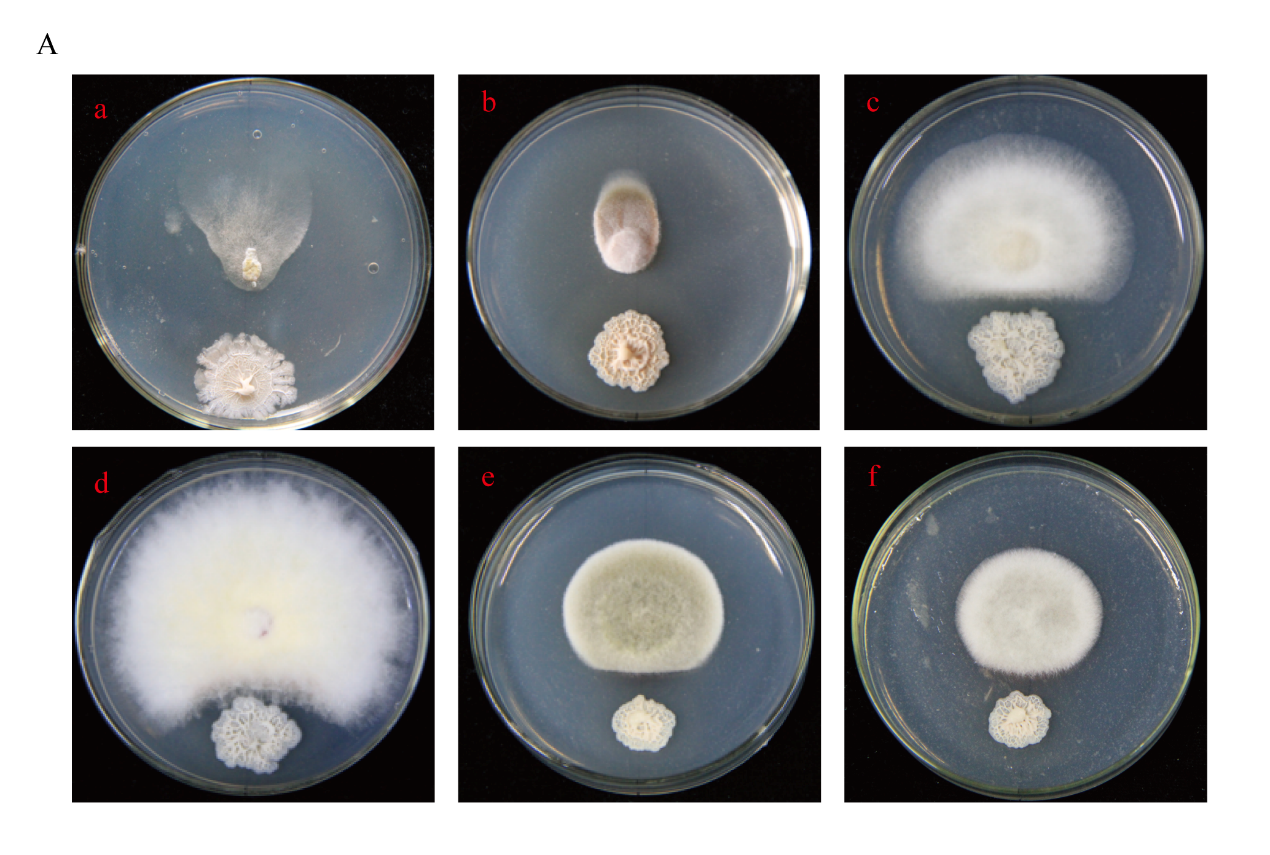


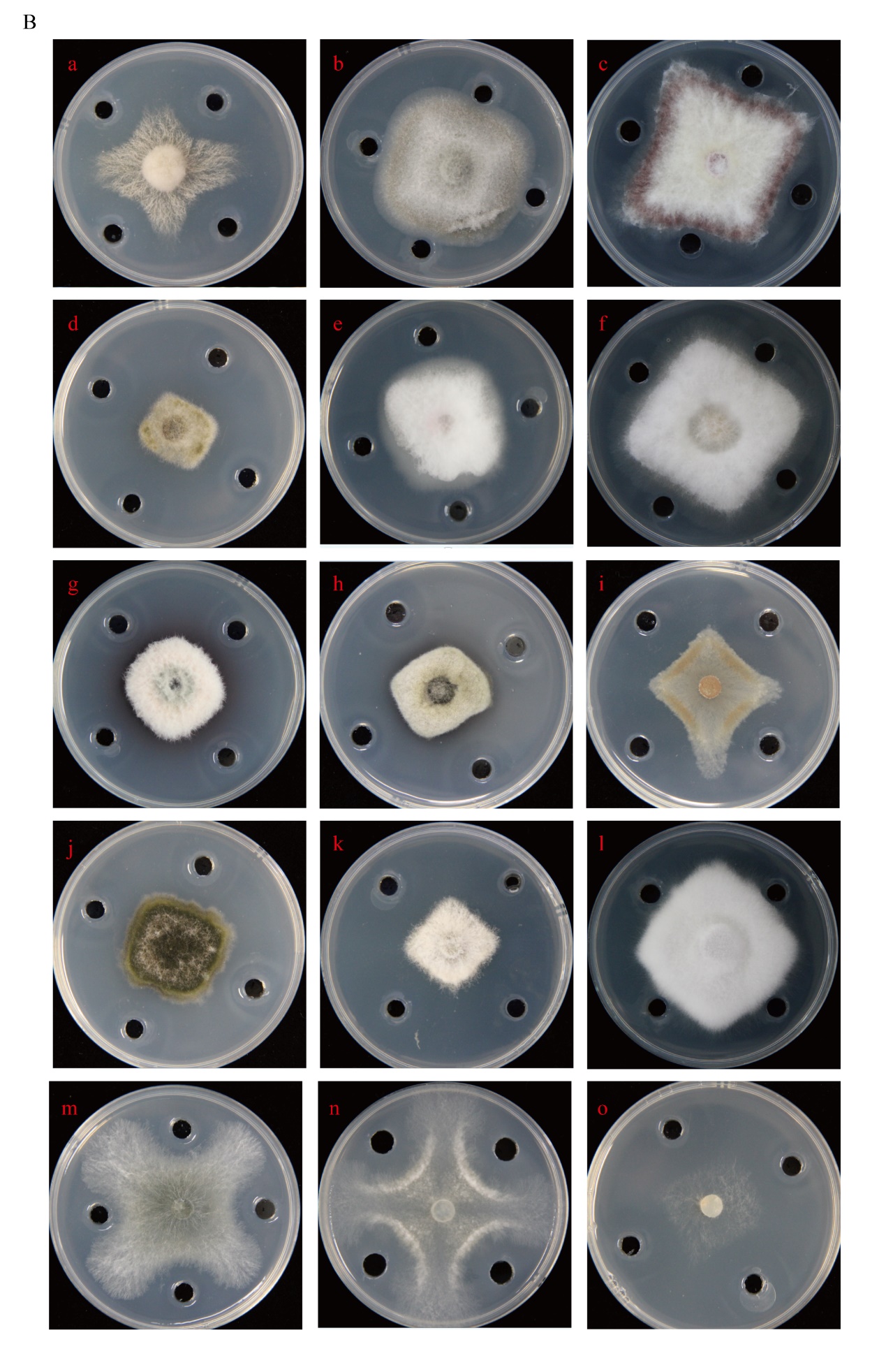


**Supplementary Figure 2.** Inhibitory effects of bacterial cells and sterile supernatants of *B. subtilis* HF1 on the mycelial growth of fungi. **(A)** The red letters, in turn, were *Trichoderma viride*, *Magnaporthe grisea* 3-2, *Glomerella cingulat*, *FusaHum graminearum*, *Alternaria alternata* (Fr) Keissler, *Corynespora cassiicola*. **(B)** The red letters, in turn, were *Magnaporthe grisea* 3-2, *Glomerella cingulat*, *FusaHum graminearum*, *Alternaria alternata* (Fr) Keissler, *Fusarium oxysporum*, *Fusarium equiseti*, *Corynespora cassiicola*, *Alternaria gossypina*, *Valsa mali*, *Alternaria alternata* f. sp. mali, *Botryospuaeria dothidea*, *Fusarium verticillioides*, *Lasiodiplodia theobromae*, *Sclerotinia sclerotiorum*, *Botrytis cinerea*. A fungal cake with a diameter of 6 mm was placed in the center of the potato dextrose agar (PDA) medium, 1 µl *B. subtilis* HF1 live cells were inoculated at the medium approximately 30 mm from the center of the fungal cake. 200 µl of sterile supernatants of *B. subtilis* HF1 was inoculated into 4 holes approximately 30 mm from the center of the fungal cake. The experiments were repeated 3 times.


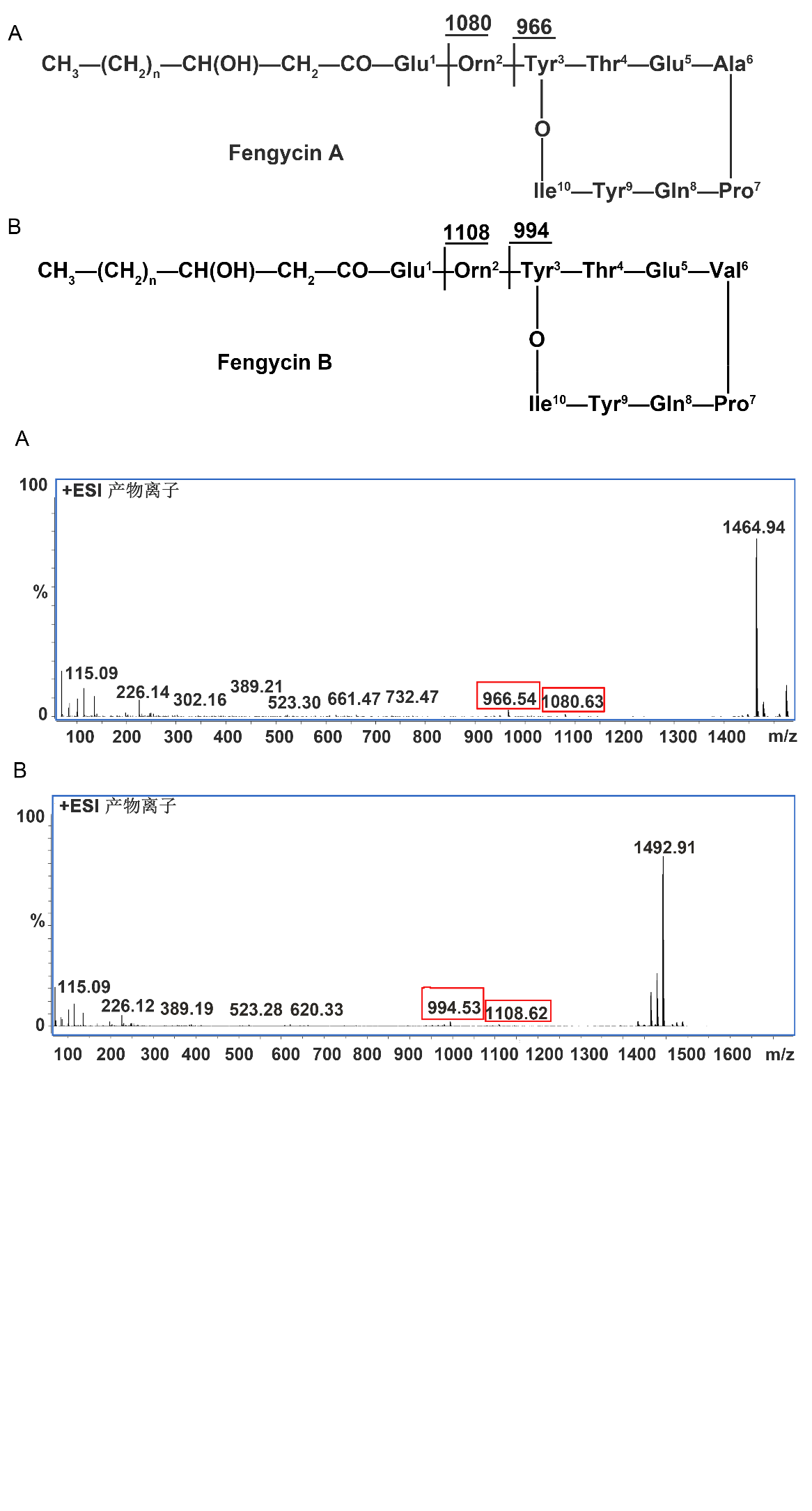


**Supplementary Figure 3.** The structure of Fengycin A and Fengycin B**. (A)** Structure of Fengycin A, the m/z 1,080 and 966 are the product ions after the partial sequence fatty acid-Glu and fatty acid-Glu-Orn are lost. **(B)** Structure of Fengycin B, the m/z 1,108 and 994 are the product ions after the partial sequence fatty acid-Glu and fatty acid-Glu-Orn are lost.

**
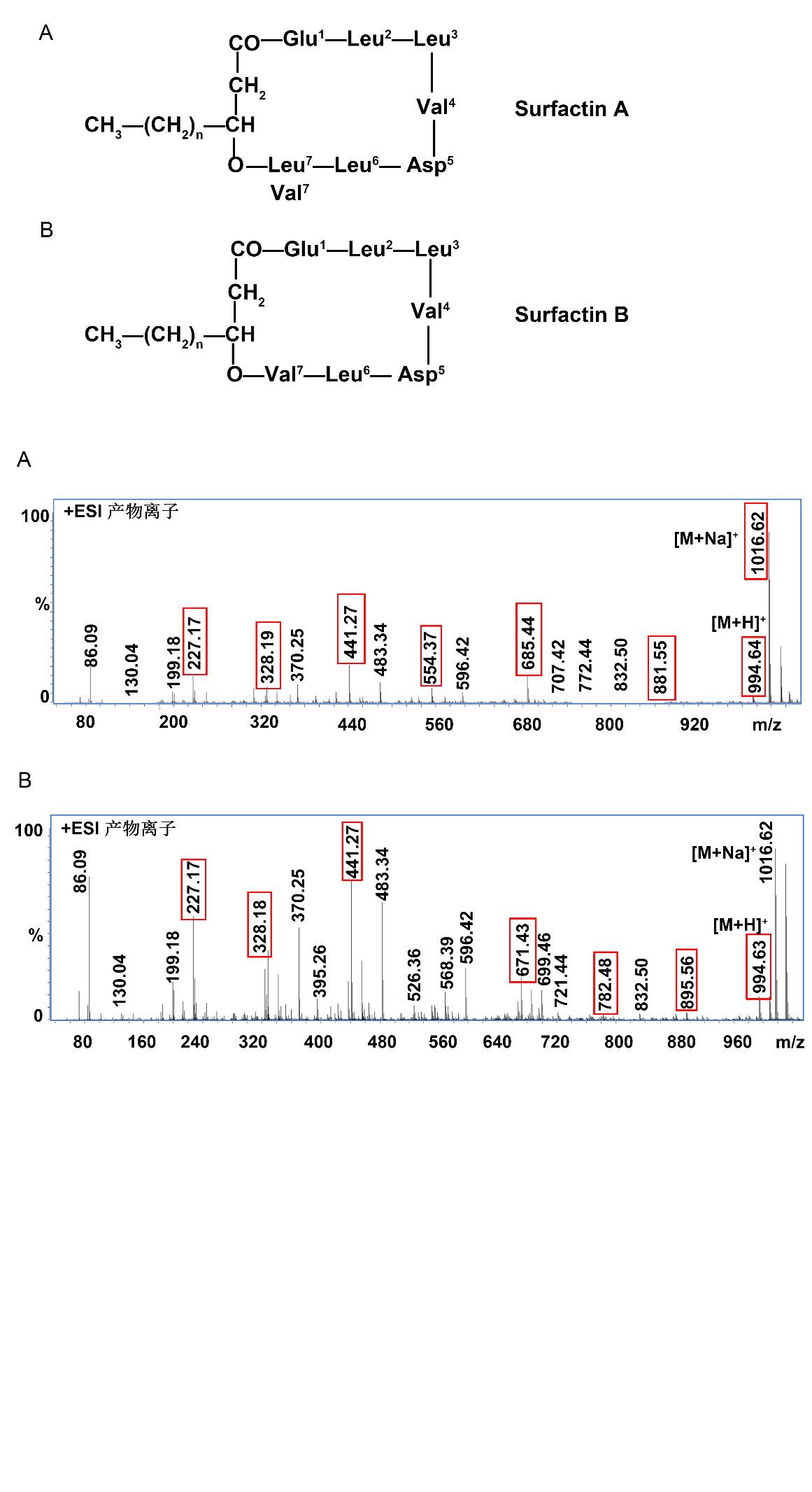
**

**Supplementary Figure 4.** The structure of Surfactin. There are two types of molecular structure of Surfactin based on amino acids at the position 7^th^ of the cyclic peptide. When the amino acid at position 7th of the cyclic peptide is Leu, the surfactin is Surfactin A, and when it is Val, the surfactin is Surfactin B. n = 9-11.

**1.2 Supplementary Tables**

**Supplementary Table 1.** Physiological and biochemical characteristics identification of strain HF1

| Physiological and biochemical tests | Test results | Physiological and biochemical tests | Test results |
| --- | --- | --- | --- |
| Glucose glycolysis test | + | D-fructose glycolysis test | + |
| Maltoglycolysis test | + | Galactose glycolysis test | - |
| Sucrose fermentation test | + | Alpha-lactose glycolysis test | - |
| Sorbitol colysis test | + | D-mannitol colysis test | + |
| D-arabinose test | + | D-xylose test | + |
| Gelatin liquefaction test | + | Starch hydrolysis test | + |
| Catalase test | +;o | Oxidase test | - |
| Anaerobic growth test | - | Voges-Proskauer (V-P) test | + |
| Nitrate reduction test | + | Propionate test | - |
| Citrate test | + | 7% NaCl test | + |
| pH 5.7 growth test | + |  |  |

The pH 5.7 growth test means that the pH of LB medium is adjusted to 5.7 to observe whether strain HF1 grows.

**Supplementary Table 2.** Comparative analysis of genomic features of strain HF1

| RefSeq Genome Features | *B. subtilis* HF1 | *B. subtilis* 168 | *B. velezensis* FZB42 |
| --- | --- | --- | --- |
| Genome size (bp) | 4,079,604 | 4,215,606 | 3,918,589 |
| G+C content (mol%) | 43.9 | 43.51 | 46.4 |
| Total number of genes | 4028 | 4354 | 3,855 |
| Average of CDS size (bp) | 891 | 895 | 933 |
| rRNA genes | 36 | 30 | 30 |
| 5S rRNA | 12 | 10 | 10 |
| 6S rRNA | 12 | 10 | 10 |
| 23S rRNA | 12 | 10 | 10 |
| tRNA | 92 | 86 | 89 |

Comparison of basic genomic characteristics of *B. subtilis* HF1, model strains *B. subtilis* 168 and *B. velezensis* FZB42.

**Supplementary Table 3.** The optimized separation procedure of surfactins and fengycins from *B. subtilis* HF1 by HPLC

| Time (min) | Water and 0.1%  trifluoroacetic acid (A) | Acetonitrile and 0.1% trifluoroacetic acid (B) |
| --- | --- | --- |
| 0 | 70% | 30% |
| 5 | 52% | 48% |
| 40 | 44% | 56% |
| 45 | 30% | 70% |
| 60 | 0% | 100% |

ZORBAX SB-C18 column (150 × 3.0 mm, Agilent), UV detection: 214 nm, column temperature: 28℃, the sample weight: 20 μL, and flow rate: 1 mL/min.
